# Evaluation of the Effectiveness of Employers and H&S Services in Relation to the COVID-19 System in Polish Manufacturing Companies

**DOI:** 10.3390/ijerph18179302

**Published:** 2021-09-03

**Authors:** Joanna Bartnicka, Patrycja Kabiesz, Dorota Palka, Paulina Gajewska, Ejaz Ul Islam, Damian Szymanek

**Affiliations:** 1 Department of Organization and Management, Silesian University of Technology, 44-100 Gliwice, Poland; Patrycja.Kabiesz@polsl.pl (P.K.); Dorota.Palka@polsl.pl (D.P.); 2 Department of Management and Transport, University of Bielsko-Biala, 43-300 Bielsko-Biała, Poland; pgajewska@ath.bielsko.pl; 3 Department of Management Sciences, Iqra University, Karachi 75500, Pakistan; eulislam@gmail.com; 4 Department of Marketing, Wyższa Szkoła Ekonomiczno-Humanistyczna, 43-300 Bielsko-Biała, Poland; dszymanek@wseh.pl

**Keywords:** safety training, didactics, self-education, safety culture, modern technology, web applications, e-learning, Moodle platform

## Abstract

With the advent of the COVID-19 pandemic, companies had to adapt quickly to survive in the market. During this time, employers played a key role, along with employees involved in Occupational Health and Safety (OHS) activities, as they were responsible for implementing the recommendations of the European Commission. There is no unambiguous definition of OHS in Polish legislation. It is assumed that it is a set of rules defining the manner of performing work, and above all, a method of providing employees with working conditions so that their performance is safe and hygienic. Responsibility for the health and safety in the workplace is imposed on the employer by the legislature. Thus, effective health and safety training is an essential element of the success of any properly operating company. In the literature, no studies have been identified that evaluate the effectiveness of actions during the COVID-19 outbreak. The aim of the article is to present the actions of Polish employers along with their effectiveness assessment related to the protection of employees during the COVID-19 outbreak. The article presents a proposal for conducting remote OHS (Occupational Health and Safety) training using the platform Moodle. The created course was implemented during OHS training conducted in a selected manufacturing company. At its end, an evaluation of the course was carried out, and the collected opinions of training participants allowed the formulation of interesting conclusions, which became the contribution of this paper. The authors pay special attention to three main points of the work. The first is the form of training, which gives the possibility to conduct training at a distance while maintaining its effectiveness. The second important point is the mandatory feedback of the trainees, ensuring the possibility of continuous improvement and quality enhancement of both the program and the form of training. The evaluation was developed on the basis of the extended Kirkpatrick model, which is a completely new approach to OHS training evaluation. The third point emphasized by the authors is the possibility of precise adaptation of the training to other plants and even industries. Therefore, it can be concluded that the course developed by the authors is a very interesting and practical didactic tool with great implementation potential.

## 1. Introduction

Safety at work is a topic of great importance and interest to both businesses and researchers [1]. Every day, people die as a result of occupational accidents or work-related diseases—more than 2.78 million deaths per year. Additionally, there are some 374 million non-fatal work-related injuries each year, resulting in more than 4 days of absences from work. The human cost of this daily adversity is vast, and the economic burden of poor occupational safety and health practices is estimated at 3.94 percent of global Gross Domestic Product each year [2]. According to Eurostat (2019a), most fatal occupational accidents occur in the construction, transport and storage, manufacturing and agriculture, and forestry and fishing sectors. In contrast, the highest number of non-fatal occupational accidents occurs in manufacturing, wholesale and retail trade, construction, and health care and social assistance [3]. Because of the high number of occupational accidents, it is necessary to improve the level of safety by creating awareness among workers. The SARS-CoV-2 coronavirus crisis has highlighted the critical importance of health, including health and safety in the workplace. The European Commission has developed a new strategic framework on occupational safety and health (Communication from the Commission to the European Parliament, the Council, the European Economic and Social Committee and the Committee of Regions on an EU Strategic Framework on Health and Safety at Work 2021–2027) [4]. This initiative builds on the previous EU Strategic Framework 2014–2020 and aims to maintain and raise health and safety standards for European Union workers. This strategic framework is particularly relevant during this extraordinary period and contains information about new risks that arise from new ways of working, new technologies and digitalization, and the COVID-19 pandemic. In addition, the framework encourages European Union member states to promote occupational safety activities, including worker training. Undoubtedly, occupational safety training raises awareness and improves workers’ competence, which is recognized as an effective way to reduce occupational accidents and improve workers’ health [5].

Improving occupational safety training in the manufacturing industry is very important due to the high number of occupational accidents and the increasing hiring of foreign workers, which creates problems related to language barriers and job instability. 

### 1.1. Effectiveness of Safety Training

**Figure 1:**
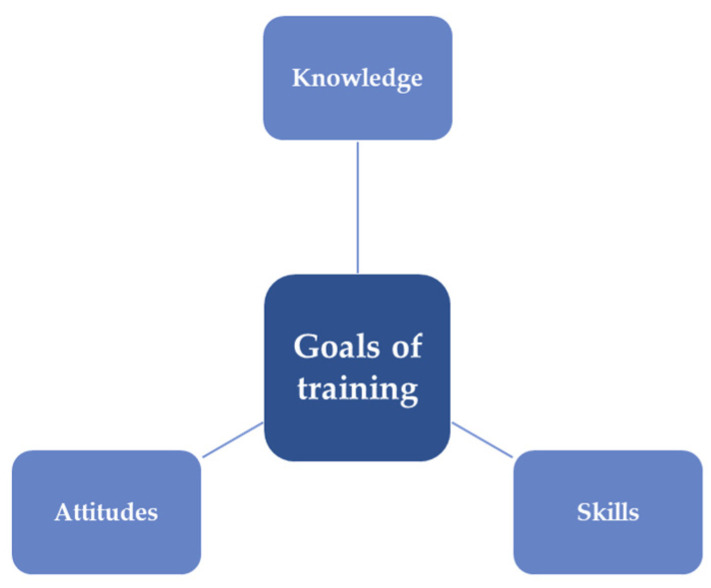
Goals of training.

Training is a planned process of changing the action/behavior of a specific group of employees built by filling the competence deficit in terms of attitudes, knowledge and skills of people, ultimately giving identifiable business benefits to the company [6,7]. There are three main goals of training: the acquisition of knowledge, skills and attitudes. The first objective refers to the acquisition of new knowledge and the expansion of existing knowledge in trainees, and the knowledge gain can be measured by a pre-training and post-training test. The second objective refers to the acquisition of skills or the improvement of existing skills, which can be seen through the change in a person’s behavior after training. Finally, the third objective refers to attitude, which is what the participant feels after the training, including motivation and self-efficacy [8,9]. Figure 1 shows the main objectives of training. 

**Figure 2:**
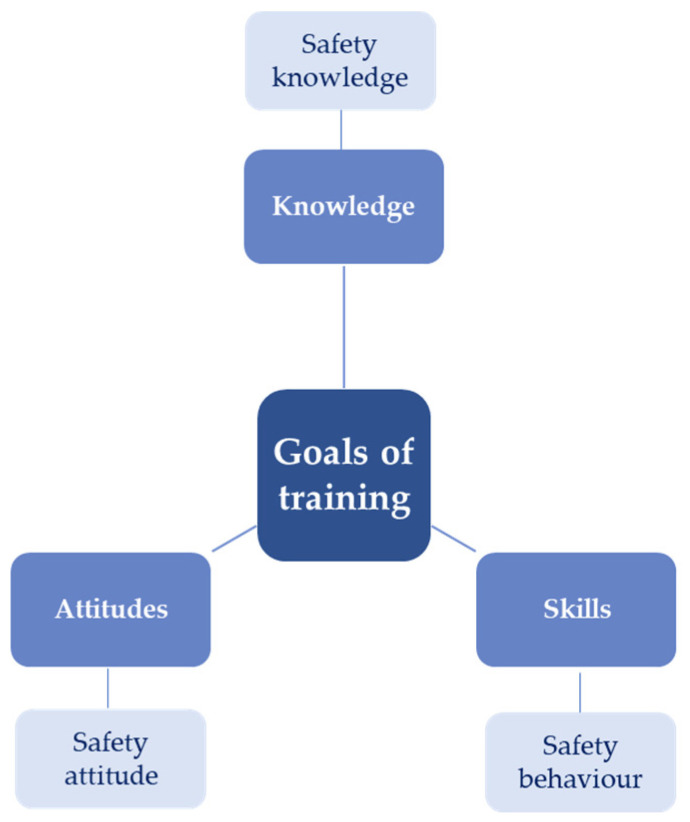
Goals of safety training.

Training is effective when it is transferred to the work environment by implementing improvements and improvement activities. Additionally, when the employee improves knowledge, skills after training and changes in attitudes in the work environment are evident [10,11]. In order to measure the effectiveness of training, Blume et al. recommended conducting training evaluation not only immediately after the training but also after a longer period of time [10]. Kirkpatrick and Kirkpatrick recommended four levels of training evaluation [12]: (a) level one: reaction, (b) level two: teaching, (c) level three: behavior, (d) level four: results, which report on the achievement of organizational goals. The first level of reaction of the participants after the training allows measuring the level of customer satisfaction, in order to get information about the attractiveness of the training. You should design the form in such a way that you encourage the post-training person to present honest feedback and suggestions in writing and get a 100 percent return of completed surveys. The second level evaluates the change in the participant’s attitude as a result of the training; there is an increase in the level of knowledge as well as an increase in the level of skills. The goal is achieved when the current attitude of the employee is changed, and the level of his/her knowledge is increased. In the third level there is a change in behavior as a result of participation in training. For such a change to occur, the following conditions must be met: the person must want to change, know what and how to do, work in a favorable atmosphere, and be rewarded for each positive change. Finally, the last level, four, is the result that brings effects and profits to the company as a result of the participation of an employee or group of employees in a training program. The results can vary depending on the type of training. In the case of health and safety, they are illustrated by a reduction in occupational accidents and diseases and a smoother flow of information between departments within the company. In addition, results can be seen through increased productivity and increased sales levels, which generates profits. It can be concluded that all occupational safety training should include three main goals: gaining occupational safety knowledge, occupational safety behavior, and safety attitudes [13,14]. This is a holistic approach to evaluating the effectiveness of occupational safety training, which is consistent with the methodology of Kirkpatrick and Blume et al. (Figure 2). 

Firstly, the purpose of safety training in acquiring knowledge about safety at work is to obtain information about the hazards present in the workplace and the necessary measures worn or kept by employees to protect him/her from one or more hazards associated with the presence of dangerous or harmful factors in the work environment (types of PPE) [15]. Moreover, during the training, the employee is acquainted with the rules of safe and hygienic work in a given establishment as well as with the work regulations, how to provide first aid, or how to proceed in case of fire (safety procedures, hazard identification) [14]. Secondly, the purpose of safety training is to shape safe behaviors, which are characterized by acting safely under risk conditions, either on a mandatory basis (safety compliance of all employees in the company) or on a voluntary basis (participation in safety) [16]. Thirdly, the training objective of safety attitudes refers to the way of behavior, patterns, aesthetic, and moral criteria adopted in a given collective [17]. Moreover, it is characterized by the involvement of employees in the formation of safety culture [18]. The effectiveness of the conducted training is influenced by the selection of an appropriate training method [19,20]. 

### 1.2. Type of Safety Training

Innovative teaching methods use motivational and activation techniques for trainees that develop skills in critical analysis of facts and creative problem solving [21,22]. Conducting trainings in such a way increases their effectiveness, as employees more quickly assimilate the knowledge and practiced skills transferred to them. The selection of training methods and resources depends on the objectives and subject matter of the training, as well as on the preferences and tastes of the trainer [23,24]. The most common methods used during safety training include [25,26,27,28,29]: (a) on-the-job training, the discovery of new capabilities of the participant, directions for the development of their competencies, setting and achieving goals, which supports the implementation of the employee to their new tasks and responsibilities in the workplace; (b) lectures consisting mainly as an oral transmission of information, useful for the transmission of new information; (c) demonstration, the demonstration or showing how to perform correctly; (d) stories and analogies, used to tell about incidents, analyze these situations and accidental events and potential accidental events in order to change the mindset of the trainees to avoid such situations again; (e) interactive lecture, when the trainer gives instructions and creates questions in order for the trainees to arrive at a certain knowledge and solution to the event or problem presented; (f) case study, when the trainer solves a specific event or situation in a planned form; (g) role-playing, the reenactment of various social interactions in order to prepare the audience so that when a real situation occurs, their reaction will be correct; (h) training games, an activating method conducted by a trainer in order to involve the participants to work out, on their own, a correct pattern of actions; (i) experiment, a very little used method in safety training consisting of achieving a very specific goal.

It is worth noting that the year 2020 (COVID-19 pandemic) proved to be a particular challenge for training professionals, as they had to find a way to transfer knowledge remotely but effectively. New methods and forms of further education and training of employees have emerged [30,31,32,33,34,35]. E-learning is a form of online training where the materials covering the scope of the training are sent to the participants. This model of training guarantees great flexibility and allows full individuality of the training in different times of access, creating flexibility and the possibility of operation of the workplace without temporary exclusion of the group of employees affected by the training. B-learning is a type of training which combines the two techniques of traditional training and e-learning. It is a particularly innovative model which is currently gaining in popularity by providing access to training materials and contact with the trainer. Webcast is a technique used by the safety department in most large corporations. It is a multimedia transmission combined with the possibility of commenting, thereby providing information for a large number of listeners. Multimedia safety kiosk is the latest development in the field of safety training. Such a device is an innovative tool for safety services, and consists of a touch screen and properly prepared application that can be modified according to changing needs. Virtual reality (VR) is used to gain experience while moving in a virtual world, in dangerous or potentially dangerous situations. Free applications can be used to create interactive quizzes to be completed live during training using a smartphone. Free applications can also be used to create evaluation questionnaires on the training, to quickly survey the training received, triggered by QR codes.

A broader definition of the term e-learning includes different forms of e-education, namely academic e-learning, school-based e-learning and corporate e-learning [36,37,38]. The school-based variety can be interpreted as a typically didactic or partially educational process, typical of primary or secondary education levels. Academic e-learning refers to higher education or training provided through university classes but using different tools, methods, means and techniques. In contrast, corporate e-learning is focused mainly on practical objectives related to improving company competitiveness, and the way of learning via the Internet can be detached from the educational institution and run spontaneously, so it could be described as non-institutional self-learning. It mainly boils down to using experience, skills, and expertise, benefiting from the knowledge of collaboration in meeting both similar and complementary needs.

The COVID-19 pandemic prompted governments, employers, employees and the general public to confront the unprecedented challenges posed by the virus and the many impacts it had on the world of work. After surveying 100 Polish manufacturing companies, as many as 80 percent of companies during the COVID-19 pandemic implemented online training via, e.g., Skype and Zoom.eu. Only two enterprises (i.e., 2 percent) conducted OHS training using the b-learning method. The remaining companies (18 percent) continued to provide training in the traditional, stationary way. Therefore, the article presents a proposal of modern solutions and methods and techniques for conducting OHS training in the remote form using the Moodle platform. The created course is a comprehensive set of teaching materials, providing high-level content and a very interesting form of learning. The proposed form of classes includes both contact with the instructor and self-education. The participant has access to lectures, presentations, quizzes, games, forum, tests and many other forms of activities. The created course was implemented during safety trainings conducted in a selected production company. At its end, an evaluation of the course was carried out and the collected opinions of the trainees allowed for the formulation of interesting conclusions.

## 2. Materials and Methods

### 2.1. Building an Safety Course Using the Moodle Platform

Currently, in the era of computerization and dynamic development of technology, the concept of e-learning is widely known. In literature, e-learning is defined as a form in which interactive computer techniques are used by a learner in a convenient place and time. In other words, it is a method of transferring and acquiring knowledge (teaching technique and learning method) using electronic media [39]. The objectives are still the same: improving the skills of employees in the context of increasing their performance. Various tools, applications and systems supporting and developing e-learning are also known. The authors have developed a proposal for a modern solution to conduct safety training in a remote form using the Moodle platform.

**Figure 3:**
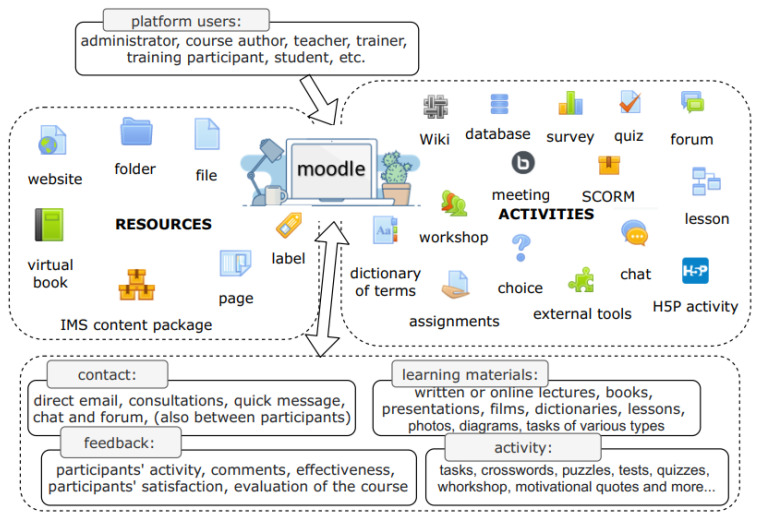
Course structure.

Moodle (Modular Object-Oriented Dynamic Learning Environment) is an educational platform that provides a robust, secure and integrated system for creating and administering personalized educational courses [40]. Its purpose is to support the didactic and methodological aspect of education. According to the source [41], the Moodle platform is successfully used in 247 countries, by nearly 271 million users. Moodle is a platform provided under an Open-Source license, so every user has access to the source code and can modify it according to their needs [42]. The construction, administration and use of the platform are simple and intuitive, requiring only basic web browser skills. The basic unit of the Moodle platform is a course, in which components in the form of resources (static educational content) and activities (activation and action-enabling content) are placed sequentially. Based on sources [43,44] and the authors’ experience, Figure 3 shows the range of available course content and basic structure.

An e-learning resource is a kind of content unit of a course, which is its substantive content. A resource can be either a teaching material attached as a pre-prepared file, e.g., PDF document, multimedia presentation, movie files, or text blocks inserted as a page, label or book content. E-learning activities are elements of the course content characterized by a certain interactivity. The components of the course that allow the instructor to take certain actions of the learner include a survey, chat, forum, quiz, or various types of assignments. In addition, all these elements building the courses can be divided by the way of communication: synchronous, ensuring contact between the participants and the instructor at the same time, and asynchronous, not requiring mutual contact at the same time. The platform provides a number of possibilities for action, which, if skillfully used by the author of the course, can contribute to a significant stimulation of learning processes and development of motivation for learning. The quality of the created course as well as its effectiveness depends largely on the intuition and creativity of the author.

**Figure 4:**
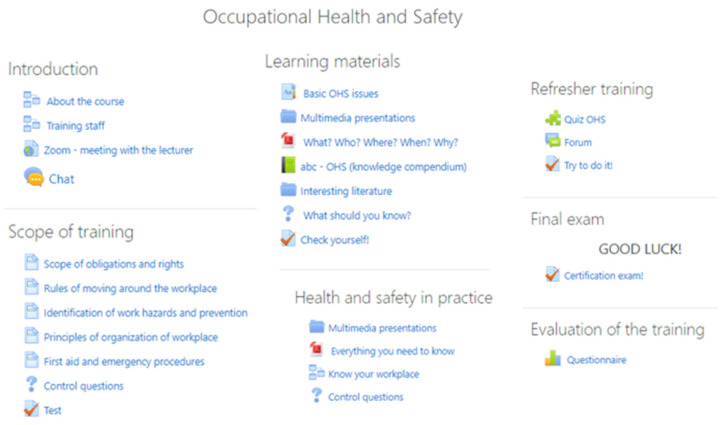
Elements of the course developed on the Moodle platform.

For the purpose of conducting occupational health and safety training in the time of restrictions related to the COVID-19 virus pandemic, a course was developed using the Moodle platform that allows the conducting of training remotely. The course was divided into seven basic parts. A pictorial structure of the course is shown in Figure 4.

The first part, “introduction”, contains information about the course and more specifically, what content it contains, what the conditions are to complete the training, how to navigate the platform, which elements are mandatory for participants, and tips on how to work on your own. Then, there is information about the trainers and a direct link to the zoom application, which enables online meetings. In addition, the possibility of communication between course participants via a chat application was made available. In the second part of the course there is theoretical material developed in the form of comprehensive lectures divided by topics. In the same part of the course there is a set of control questions that enables self-verification of knowledge. In addition, the participants have the opportunity to check themselves using a test that each time generates 5, 10 or 15 random questions from a selected topic or the whole material. The third part of the course includes “learning materials-theoretical information” aimed at broadening the knowledge of occupational safety and health. The materials have been prepared in various forms: pdfs, videos, presentations, external links, collections of articles, books, and glossaries. The diversity of the developed training content arouses interest and has a mobilizing effect on participants. The fourth part of the training, which includes practical examples of OHS in a real company, was developed in a similar way. For training in a food processing company, materials were prepared with regard to this industry. The fifth part of the training includes the most interesting form of learning (in the opinion of the authors) which includes developed tasks in the form of interactive games, puzzles, crosswords, and riddles. The sixth stage of training includes a final exam, which covers the material of the entire safety training. The authors of this work have taken full care to prepare a comprehensive database of questions taking into account the guidance of experts, the results of scientific research, and their own teaching experience. A database of approximately 300 questions was prepared from which, during the exam, the computer draws 25 questions for the examinee from five thematic groups. Time to complete the exam was limited to 20 min. The questions are closed tasks: multiple choice (a, b, c, or d), true/false, matching in pairs, drag and drop (drawings or texts), missing word choice. The authors deliberately omitted questions of an open-ended nature in order to avoid subjective evaluation of the answers given. Moreover, the nature and form of questions take into account the problem of non-self-directed work of remote training participants. In open-ended questions you will very often find pasted answers or fragments of answers from available sources. Closed questions and time pressure limit the possibility of non-self-dependent work. Assessment of the acquired knowledge is carried out in the mode of electronic verification of the compliance of answers in the system. Immediately after confirming the entered answers, the participant receives feedback on the correctness of the answer (acceptance or rejection of the result by the system). The user also receives a complete point and percentage analysis along with the final grade for the work completed. In the last part of the developed course there is an evaluation component based on the Kirkpatrick method. The evaluation process is described in detail in Section 2.3.

### 2.2. Occupational Health and Safety Training

**Figure 5:**
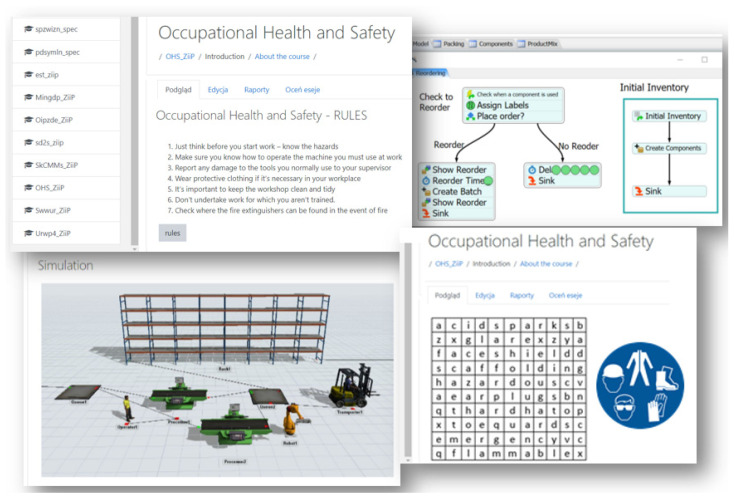
Examples of the developed content and tasks of safety training.

The created course was implemented during health and safety training conducted at a selected manufacturing company. A total of 124 employees participated in the training. The training began with a test to verify knowledge of occupational health and safety. The duration of the training, from enrolling participants to obtaining certificates, was not limited in any way. This means that a participant in occupational health and safety training can solve the mandatory tasks and take the final exam at any time. Obtaining the certificate entitles them to work in the company. The course participant has the opportunity to prepare for the final exam independently and effectively by studying specially prepared materials and solving prepared tasks. Examples of the learning materials are shown in Figure 5.

Due to the ever-changing market and work environment, the development of employees’ competencies and skills is an essential part of the functioning of modern companies [45]. Therefore, the developed training materials have an activating and engaging character, which helps to overcome the resistance to assimilate new knowledge. It should be remembered that this is a common phenomenon in adults, unlike adolescents or children [46]. The content was presented in a clear, readable and understandable way for the participant. The difficulty level of the tasks increases gradually according to the course of the training. In addition, the theoretical content conveyed was presented to the real working conditions in an enterprise of the same industry. Tasks in the form of interactive or simulation games are designed to consolidate the newly acquired knowledge and new habits of safe attitude, as well as the necessary rules of behavior at a given workplace [47,48]. One of the tasks aimed at familiarizing employees with chemical hazards in the work environment, as well as in everyday life, was a game of matching a warning pictogram (chemical substance label) with a description and sample graphic of a chemical substance. Through additional exercises of this type, trainees practice perceptive memory and reflexes, concentration skills, as well as consolidate the necessary theoretical knowledge [49,50,51,52,53,54]. Thanks to this, on the shop floor they will very quickly spot and recognize pictograms, so they will know the hazards that occur at their workplace. One of the additional tasks was an application allowing the arrangement of virtual puzzles which contain information about noise. Puzzles are not only an element of entertainment, but also play an educational role, because we can find in them information about the effects of exposure to noise, types of personal protection equipment, and the noise levels generated by the surrounding sound sources.

The course was supervised throughout by presenters, who provided ongoing support in solving both technical and substantive problems. All possible channels of communication were made available, both with the trainers as well as with other participants of the course. Most participants contacted each other through direct quick messages platform, and less so through email. The forum and chat enjoyed great interest and involvement of participants. In addition, online lectures of the training content were conducted, allowing synchronous meetings with the trainer and other trainees. These meetings were very popular, especially working in groups called “breakout rooms”. For those interested, consultations were held, during which there were discussions and problem solving. Thanks to the great involvement of listeners, very difficult and ambiguous situations were solved, which proved to be a very interesting and useful element of training.

### 2.3. Course Evaluation

The final section of the developed health and safety training course included an evaluation component. It consisted of a questionnaire survey of the participants in order to obtain an evaluation of the developed training content, the form of its implementation, the satisfaction, and general opinion of the participants, and the effects and profits of the company. The evaluation was performed using a questionnaire based on the Kirkpatrick method. Two evaluation questionnaires were made available, the first one, intended for trainees, was divided into three levels, the second one, intended for management and executives, included only evaluation of training results in the form of company effects and profits. The research sample in questionnaire one was 124 employees working in a manufacturing company, and in the second was the management and directors of the plant in the number of 12 people. Both questionnaires obtained 100 percent return of the questionnaires.

Questionnaire one included the following stages:

1. Response—The first stage of the questionnaire contained five questions to measure the satisfaction of the trainees using a 7-point Likert scale, where 1 meant the lowest rating and 7 meant the highest rating. The questions concerned the attractiveness of the developed course and the training techniques used, including tools and mixed b-learning technique (independent work on the platform and online classes and consultations through Zoom).

**Figure 6:**
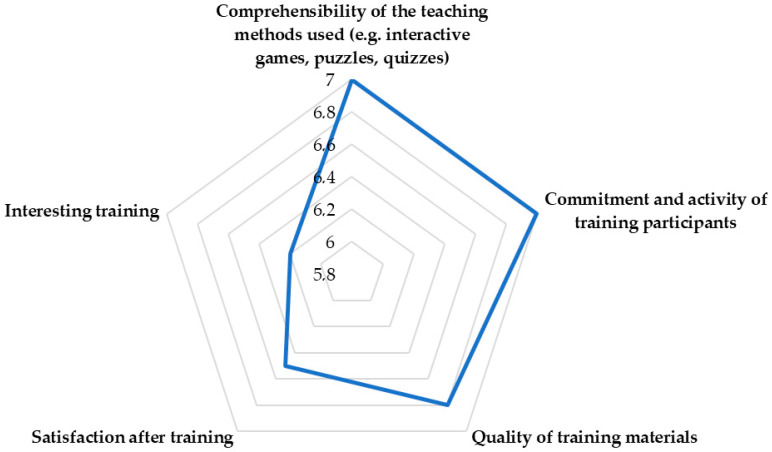
Assessment of the satisfaction of the respondents after the training.

The responses obtained showed that all respondents rated the attractiveness of the training and the involvement and activity of participants in the training at the maximum number of points. Figure 6 shows the average score from the responses obtained by the respondents after the training.

**Figure 7:**
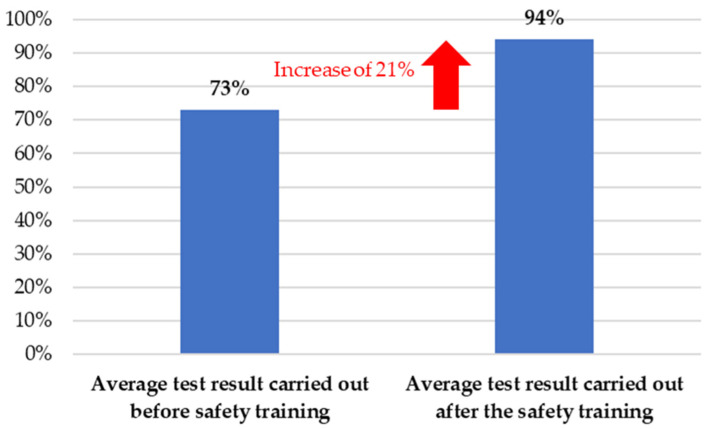
Average result of the test of knowledge of occupational safety rules by employees.

**Figure 8:**
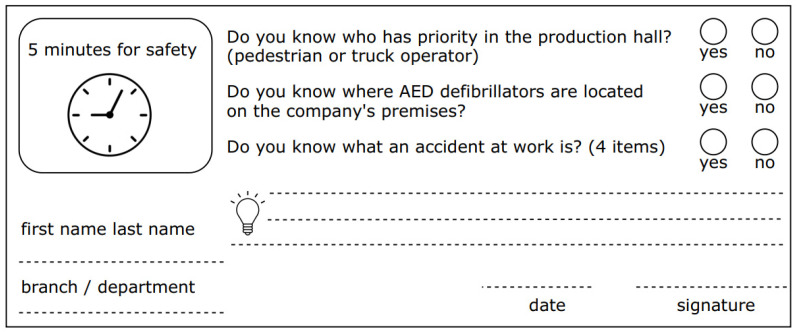
Sample questionnaire “5 min for safety”.

2. Learning—In the second stage of the survey, the results of the initial test and the final exam were compared to determine the level of increase in knowledge. In addition, participants were asked to provide candid comments regarding their feelings after receiving the training. Figure 7 shows the average of the pre-training and post-training workplace safety knowledge test scores. It is worth noting that the knowledge of the employees on workplace safety where the study was conducted is at a high level. After the training, the level of knowledge among employees increased by 21 percent.

3. Behavior—The third stage of the survey refers to the changes that occur in the employee’s behavior. This change is related to the employee’s awareness of their knowledge and the form of compensation (mobilization). The behavioral changes of the trainees were verified by conducting moderated conversations on the topic of job security between the supervisor and the subordinates. These meetings are called “5 min for safety” and were held at each work shift once a week, during which employees had the opportunity to discuss problems occurring during work tasks using a brainstorming technique that stimulates creative thinking. Employees in team groups develop ways to solve problems and then the best solution is selected. At the end of the meeting, each worker received a questionnaire with several questions about safe work. An example of this type of questionnaire is shown in Figure 8. Each week, employees answered different questions, and if a subordinate was unsure or unaware of a particular topic, the manager conducted a follow-up interview immediately after completing the questionnaire.

4. Evaluation results—The fourth stage of the evaluation, which involved assessing the effects and gains to the enterprise that have occurred as a result of an employee or group of employees participating in the training program, was provided in a separate questionnaire. The second questionnaire was prepared for the company’s management and executives, those who can assess the awareness and perceptions of employees based on events occurring in the workplace. Initially, a month after the training was conducted, the questionnaire was completed by 12 of the plant’s management members. However, it turns out that this is too short a period of time to observe changes. In addition, it was determined that after such a short break since the training was completed, the results may be inflated due to the “fresh eyes” of the employees. It was decided that the last part of the evaluation would be repeated after a six-month break from the training date.

## 3. Results

In the evaluation phase of the training 124 respondents took part, including 53 women and 71 men. The most numerous groups of respondents were those falling within the age range of 36–45 years and 26–35 years, while the least numerous groups were those over 55 years of age. The age of the respondents seemed to be an important issue in relation to new technologies and educational methods. It turns out that older people were reluctant to use the e-learning platform, most often preferring traditional methods of learning in the form of provided written materials with the possibility of printing [14,55]. The greatest interest in the posted learning activities was shown by the youngest group of respondents in the age range of 18–25 years. It should be remembered that these are young people, accustomed to modern solutions used in schools or universities. At the same time, these people are very critical and demanding, because they have a lot of experience with modern Internet applications and tools. On the other hand, these people have no professional experience because they are just entering the job market. Therefore, the content provided in the field of occupational safety and health must be prepared accurately and its effectiveness should not overshadow the essence.

Among the participants, people with higher education dominated, constituting 73 percent of the group; 17 percent of the respondents had secondary education, 5 percent had post-secondary education, 6 percent of the respondents had basic vocational education. People with primary education did not participate in the training. It can be noticed that people with higher education were more active on forums and during online meetings. However, the analysis of the survey results did not show a clear distribution of participants’ preferences based on their education.

Among the provided didactic materials, the respondents valued the interactive simulations as well as films and presentations containing photos of the company. The possibility of unlimited access to sample tests verifying knowledge turned out to be important for participants. Over 90 percent of trainees solved additional quizzes, control questions and self-tests at least three times. The course participants did not show any interest in tasks such as crosswords, cross-outs or rebus puzzles. Puzzles were considered a curiosity and a form of relaxation, but they were not interpreted as didactic forms.

One of the analyzed issues was the level of knowledge after the training, determined by the trainee. Most participants indicated their level after training as very good (86 marks), then good (34 marks) and average (4 marks). None of the participants marked their level of knowledge below the average score.

Responses regarding satisfaction with the training varied. It appears that younger and educated people showed more engagement and rated the training as satisfying. Respondents of older age groups with work experience were not satisfied due to the need to work independently. Most of them were those who were joining the training only because of the legal requirement.

The last stage of training evaluation concerned the changes that occur in an enterprise as a result of training. The questionnaire respondents were 12 representatives of managerial positions and company management. Among respondents there were four women and eight men, and all of them had higher education. The survey took place one month after the training was completed. No accidents and near misses were recorded during this period. There was also a decrease in the rate of accident incidents, which are undesirable events that do not cause injury to workers but only damage to property or loss of production. However, due to the short time since the training was completed, these results may seem unreliable. In addition, employees during this time exhibit what is called “fresh eyes” which can definitely understate the results.

## 4. Discussion

The dynamic development of technology, electronics and the Internet, among other things, has contributed to tremendous changes in the processes and ways of acquiring knowledge, competencies and social skills and widely understood personal or professional development [13,19]. In the field of education and training, there appears a widely understood and constantly developing concept of e-learning, which can be simply explained as distance learning. Its advantages usually include flexibility and mobility, i.e., the possibility of conducting the educational process at any time and at any place [18,20]. Moreover, a lack of territorial limitations is mentioned as one of the most important advantages. This means the possibility to participate in training courses organized by educational units, universities, training companies, and companies or other institutions located anywhere in the world. Breaking the spatial barrier is an ideal advertisement and a chance to gain a wider group of supporters, clients or students.

Remote teaching is characterized by a special specificity. It results primarily from the need for the user to act independently and communicate non-verbally with the instructor (mostly). Participants of safety training are most often adults taking up a job in a selected company or enterprise. Depending on the industry, as well as the position being undertaken, these individuals vary in terms of perception, IT skills, and work experience, as well as stress levels, learning and mobilization skills. Some of these people are unable to adapt to b-learning by encountering a technical barrier related to the need to operate a computer, a remote learning platform, and the applications contained therein. This barrier automatically generates a sense of stress, thus lowering the trainee’s mobilization level. For such people it is recommended to organize additional consultations individually or in small subgroups. Most often they rely on technical assistance, i.e., explaining the principles of operation of the platform, applications and even individual windows or tasks. It is worth remembering to choose a trainer who will show patience and creativity in communicating information. An important element here is to control the growing stress and frustration of users and encourage them to use modern technologies.

In other cases, the necessity of acting independently can generate non-technical factors, which include a lack of mobilization to learn, aversion to computer applications, difficulty in concentration, lack of consistency, and the phenomenon of procrastination. In such cases, automatic notifications reminding you to log in or perform a task are useful. Most often these are automatic e-mails containing information about deadlines, progress statistics and mobilizing content. Among the participants of remote training, it can also be mentioned the people who have difficulty in finding themselves in written communication. A common phenomenon of remote training is lack of knowledge of network etiquette (netiquette), especially visible in forums and chat rooms. Comments from participants are often peppered with emoticons in an attempt to replace non-verbal signals such as smiles, anger or surprise. Comments also tend to be uncensored or vague and redundant. Therefore, it is an extremely important part of training to establish and develop appropriate communication norms and rules [56]. Thus, it is obvious that remote training participants should not be left without the support and supervision of trainers. Remote contact (Zoom, MS Teams) may not replace real contact in the real world, but the support that can be provided to participants solves most of the described problems.

As mentioned earlier, participants in safety training belong to different social groups. Their ability to assimilate knowledge is shaped at different levels. For this purpose, clear and readable training materials as well as activating and engaging tasks were developed. Interactive games are a form of fun, in which the user does not feel time pressure, pressure of new information or boredom resulting from the monotony of the activity. Moreover, exercises were divided thematically, with the possibility of choosing the level of difficulty. A great advantage of remote training is the ability to adjust the pace of work to individual units. It allows for conducting training fast with people well acquainted with the material, and slow with a part of the group that needs time to assimilate the content.

By the effectiveness of the training, we partly understand the final results obtained by the participants completing the final exam. To a large extent this result depends on the participant’s interest, involvement and willingness. During remote classes the possibility of forcing trainees to learn is limited. This means that the learning materials must be prepared in such a way that they fully interest the participants. Due to the diversity of trainees, this is a difficult task. There will certainly be supporters of colorful interactive multimedia presentations, as well as supporters of traditional forms of learning in the form of a book or a modest written study. Preferences should be considered both in terms of the materials provided and the tasks to be completed. A general good practice to attract trainees is to provide news, trivia, and practical examples in an easy-to-understand form. According to the authors’ research [10,25], most prefer modern training techniques and a short and concise form of communication.

The disadvantage of remote training perceived by the authors is the lack of integration of the group. Participants remain anonymous to each other and do not establish relationships as during traditional stationary meetings. Few people turn on the webcams so that they are visible during the classes, the vast majority remains in off-camera mode and microphone mode. From the point of view of the trainer, lecturer or coach, this makes it difficult to conduct online classes. Participants are reluctant to answer loosely thrown questions, it is difficult to start a discussion with the listeners, and it is also difficult to use the brainstorming method. In addition, another disadvantage of remote training is the difficulty in conducting workshops and laboratory classes. Not all types of workshops and laboratory classes can be conducted in the virtual world, for example, chemical experiments on determining the properties of a designated substance, workshops on operating fire-fighting equipment, or conducting a trial evacuation of people on the premises.

Another disadvantage of remote training perceived by the authors is the problem with the verification of knowledge. The remote form of training facilitates the participant’s unassisted work. A common phenomenon is participants sending each other ready solutions of tasks, screenshots of tests and even examination questions. In the majority of open questions, one can expect a pasted answer or its fragment from available papers or websites. Therefore, exams conducted in a remote form should be carefully thought out and refined.

Currently, in the era of dynamic technical changes, the level of competitiveness of companies and enterprises on the global market is increasing. Thus, there are numerous changes in the work environment, which create the need for periodic training. Raising skills and competencies of employees, increasing their awareness of existing hazards and the elimination of these hazards is the basis of properly conducted safety training. Thus, the approach to the interpretation of the notion of training effectiveness has changed. This effectiveness no longer means only a satisfactory level of passing the final exams, but also the ways of conducting classes, and the techniques, methods and tools used. Therefore, an important element of a course is its evaluation, understood as participants’ feedback. On the basis of obtained opinions, comments and evaluations, it is possible to improve those elements of training which participants (in the meaning of recipients) indicate as burdensome, boring or incorrect. On the other hand, it should be remembered that traditional stationary training has to be adjusted to the group (so called majority) of participants, and not to individual units. In the case of remote training, such modification is possible, but requires additional involvement of the course instructors or authors. In the opinion of the authors of this paper, the remote form of training seems to be more flexible and oriented to the needs of the participant.

## 5. Conclusions

Although the b-learning method of conducting training is well known, it is still not often used in Polish manufacturing companies.

The aim of this publication was to present the actions of Polish employers along with their assessment of the effectiveness related to the protection of workers during the COVID-19 epidemic. The article presents a proposal for modern solutions for conducting OHS training in the remote form in response to the restrictions associated with the COVID-19 virus pandemic. The authors focused particular attention on three main points of the paper. The first is a modern form of training that provides the possibility of conducting training remotely while maintaining its effectiveness. The second important point is the feedback from trainees ensuring the possibility of continuous improvement and enhancement of both the program and the form of training. The third point is the possibility of precise adaptation of training to other companies or even industries. Thus, we may conclude that the course developed by the authors is a very interesting and practical didactic tool with powerful implementation potential.

Based on the analysis of the literature on the subject, the authors’ experience and the analysis of the survey results, the following conclusions have been drawn:

1. The most important advantages of remote training include: no time and territorial restrictions; financial savings; the possibility of personal development in a flexible form; the possibility of better and more effective management of study and work time; learning new techniques, tools, applications; development of independence, creativity and self-discipline; individual pace of work and way of learning; and the possibility of quick and efficient modification of the content of educational resources.
2. Among the most important difficulties and limitations can be listed: technical barrier, difficult contact (mostly written), lack of real contact between trainees, lack of relationship and integration of group, difficulty in maintaining constant activity of participants, difficulty in maintaining adequate motivation for learning, a large workload of training and course authors, the need to have extensive knowledge of various disciplines, difficulty in verifying the real knowledge of trainees, and the common phenomenon of copying answers, downloading and using additional sources.
3. The first stage of the questionnaire showed that participants particularly praised the teaching resources in the form of simulation videos and presentations containing photos of the company. Quizzes and self-tests to verify knowledge were among the most appreciated activities. The trainees did not show any interest in crosswords, rebus puzzles, and punch lines.
4. As a result of post-training evaluation, almost 70 percent of trainees assessed their level of OHS knowledge as very good, and almost 30 percent as good.
5. Changes in employee’s behavior are related to their awareness and knowledge and form of remuneration (mobilization). These changes were verified by conducting moderated conversations on the topic of work safety between the supervisor and the subordinates. In addition, a survey verifying the so-called “5 min for safety” was implemented.
6. The results of the effects and gains obtained as a result of the training will be reassessed 6 months after the training to ensure their credibility.

Despite the numerous drawbacks and barriers that can be demonstrated against a remote form of training, the course developed by the authors provides a very interesting and practical teaching tool with powerful implementation potential that can be used in the next wave of COVID-19 cases.

## Data Availability

The data presented in this study are available on the reasonable request from the corresponding author.
